# Policy implications of big data in the health sector

**DOI:** 10.2471/BLT.17.197426

**Published:** 2017-11-23

**Authors:** Effy Vayena, Joan Dzenowagis, John S Brownstein, Aziz Sheikh

**Affiliations:** a Department of Health Sciences and Technology, ETH Zurich, Auf der Mauer 17, 8092 Zurich, Switzerland.; b World Health Organization, Geneva, Switzerland.; c Department of Biomedical Informatics, Harvard Medical School, Boston, United States of America.; d Usher Institute of Population Health Sciences and Informatics, University of Edinburgh, Edinburgh, Scotland.

Over the last decade, there has been growing enthusiasm for data analytics as well as growing appreciation of the potential usefulness of so-called big data in transforming personal care, clinical care and public health, and related research. Both the public and private health sectors are investing in the technologies and analytical capabilities needed to unlock the full value of big data. For governments that are interested in using such data, a natural starting point is to link national health-care data sets, to facilitate in-depth analysis of the performance and utilization of health services. At the institutional level, the analysis of electronic health records may greatly expand the capacity to generate new knowledge by creating an observational evidence base to help resolve clinical questions.^1^ Analysis of big data is already proving critical in building accurate models of disease progression and providing personalized medicine in clinical practice. It has also facilitated the evaluation of the impact of health policies and improved the efficiency of clinical trials.^2^ By encouraging patients to participate in their own care, delivering personalized information and integrating medicine with behavioural determinants of health, the integration of electronic health records with personal data from other sources, e.g. medical devices, wearable devices, sensors and tools based on virtual reality, could also be very beneficial.^3^ The value of health research based on non-traditional data streams from Internet-based applications, platforms, e.g. social media and services, e.g. email and online purchasing, has already been demonstrated. For example, during the Zika virus outbreak in 2015, analyses of reports in the online media helped to supplement existing information, close knowledge gaps and allow researchers to estimate transmission dynamics and plan response measures that extended beyond vector suppression.^4^

## Big data ecosystem

**Fig. 1:**
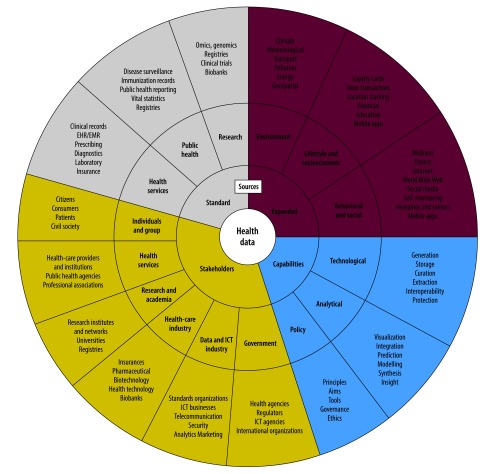
The evolving data ecosystem that links health-related big data, 2017

EHR: electronic health record; EMR: electronic medical record; ICT: information and communications technology.

Note: The figure summarizes the three main feature of the ecosystem, i.e. the expanding sources of health data, the increasing capabilities that enable data investigation and use and the diversity of stakeholders, that, together, are creating new opportunities for health.

Source: Adapted from ^5^

New opportunities are being opened by the continuing expansion of the possible uses, sources and types of big data. For big data on health, the stakeholders extend beyond health-care providers, patients and research institutions to include businesses, development agencies, national governments, professional societies and other entities that are not necessarily directly related to health research or the delivery of health services. As new analytical models, data sources and stakeholders increasingly build into dynamic relationships, it may be helpful to think of health-related big data as an evolving ecosystem (Fig. 1).^5^ There are several challenges to the future development of this data ecosystem. Governments need to consider how to reshape national policies to advance the use of big data in health while keeping such data confidential, private and secure. Risks may arise not only directly, e.g. from the characteristics and scope of the data, but also indirectly, e.g. from the ways in which the data are combined, the policies, systems and technologies used to manage the data and the ways in which the data may be used. Even basic health data can be misused and lead to discrimination, especially of vulnerable populations. The fair distribution of any new benefits that may arise from the collection and analysis of big data may also pose hard challenges.

## Big-data approaches

Despite increasing awareness of the benefits of big data and the related methodological and technological advances that are being made, many countries appear to be slow in adopting approaches based on such data.^6^ The reasons may include gaps in funding, leadership and technical expertise and competing priorities within the health system.^7^ Many governments are still considering appropriate policy options. In 2015, according to the World Health Organization’s Global Observatory for eHealth, only 21 (17%) of the 125 Member States surveyed reported having a policy or strategy regulating the use of big data in their health sectors.^8^

## Policy directions

Any government that adopts big-data methods and technologies in the health sector will need to establish proactive and durable policies to protect the health data of individuals, i.e. in terms of confidentiality, privacy and security, tackle the probable pressure for the commercialization of the data and promote the interoperability and use of the data for the public good. We believe that the focus should be on three priority areas: access and benefit sharing; accountability and transparency; and quality and safety.

### Access and benefit sharing

The full beneficial exploitation of health data is dependent on the data being accessible to those who will use them for the public good. Such access requires a comprehensive framework for data governance that defines the conditions of data access, including appropriate safeguards, the responsibilities and roles of data users and the principles of benefit sharing.^9^ In the past, such frameworks generally placed most ethical control of data use at the point of data generation, i.e. via the collection of informed consent from patients or care providers. However, in the context of big data, ethical control may need to be exerted for many years after the data have been collected and whenever the data are used.

The use of effective and proportionate privacy safeguards can facilitate data access. Legal reforms on data protection and privacy and are underway in many countries. For example, the European Union has adopted a General Data Protection Regulation and Switzerland is drafting a revision to its Federal Data Protection Act. Such reforms attempt to increase the data subjects’ privacy options and introduce further controls on data uses. However, the control of access to data needs to cover not only data protection, but also the distribution of any benefits of the exploitation of personal data and the public acceptability of such exploitation.^10^ Provisions for fair benefit sharing need to form an integral part of any policies on data access. Such policies need to be citizen-centric. Citizens are increasingly demanding access to their own data, partly to control the secondary uses of the data and partly to use the data themselves.^11^ Technology, such as the internet can facilitate public access to health data and citizens who seek such information online can provide useful indicators of emerging health threats.^12^ Citizens are important stakeholders in the development, evaluation, implementation and monitoring of data initiatives. Their role should extend far beyond the provision of informed consent for data use and include involvement in the governance of data initiatives and negotiations on the fair sharing of the benefits of data exploitation.

### Accountability and transparency

The increasing complexity and sophistication of data-linkage methods pose a risk of making data transactions more opaque. This is especially relevant in the health sector, where accountability and transparency are critical in the development and maintenance of trust. Policies on data management need to focus on maintaining a high level of transparency through engagement in iterative processes that involve all relevant stakeholders. Over time, the traditional actors in the health sector have developed accountability processes that now need renewal, revision and extension to include new actors, e.g. data and social media companies and new capabilities, e.g. predictive algorithms based on artificial intelligence and cover everyone involved in the handling of personal data. In the control of big data, sound accountability mechanisms can help in monitoring compliance with privacy protection and other ethical norms and fairness in benefit sharing and they can also provide avenues for seeking appropriate remedies in the case of failure. As predictive algorithms find their way into future health care, it may be difficult to determine how earlier decisions have been made. In anticipation of these developments, policies should demand that accountability, the critical questioning and monitoring of algorithmic decisions and the evaluation of the consequences of such decisions for care seekers are all integral parts of any system that involves health-related big data.

### Quality and safety

Any information systems where health data are generated, stored, managed and used need to adapt continuously to meet evolving quality and safety standards. Policies can either mandate or provide incentives for the standardization and system optimization needed for such adaptation.^13^ At national level, effective strategies for data integration, interoperability and security standards are essential if data safety and security are to be assured. By setting standards, the engagement of industrial stakeholders can be promoted and both competition and quality can be enhanced.

## Conclusion

In the field of health-related big data, the public needs to be reassured that security measures are mandated and enforced. Policies can, and should, address the adoption of appropriate technologies, the evaluation and monitoring of security systems and accountability and transparency mechanisms, e.g. legal remedies and compensation for those harmed by security breaches. Data security, as a societal and technological norm, will continue to evolve while the big-data approach demands more regulatory oversight, responsive policies and technical skills.

Future policies must take into account the distinct challenges posed by big data as well as the potential benefits. They also need to be applicable to the full range of stakeholders, not least to the general public and must be accompanied by a level of accountability that, over time, is sufficient to maintain the public’s trust and confidence in data usage.
